# Complete chloroplast genome sequencing of *Pseudocodon convolvulaceus*, a medicinal herb from Qinghai-Tibet Plateau in China

**DOI:** 10.1371/journal.pone.0328307

**Published:** 2025-08-18

**Authors:** Likuan Liu, Qiwen Li, Jingxuan Du, Weibo Yuan, Rui Sun, Haoyu Liu, Jinping Li

**Affiliations:** 1 Qinghai Provincial Key Laboratory of Medicinal Plant and Animal Resources of Qinghai‒Tibet Plateau, School of Life Sciences, Qinghai Normal University, Xining, Qinghai, China; 2 Academy of Plateau Science and Sustainability, Qinghai Normal University, Xining, Qinghai, China; Kerman University of Medical Sciences, ISLAMIC REPUBLIC OF IRAN

## Abstract

As a medicinal plant on the Qinghai-Tibet Plateau, *Pseudocodon convolvulaceus* has garnered significant attention due to its rich medicinal value, demonstrating notable anti-inflammatory and antioxidant activities. To elucidate the characteristics of the chloroplast genome and the phylogenetic position of *Pseudocodon convolvulaceus*, as well as to explore its genetic structure and evolutionary significance, the complete chloroplast genome was sequenced, assembled, annotated, and compared with the published genomes of the Campanulaceae family. This analysis provides insights into gene content, structural variation, and phylogenetic relationships. A phylogenetic tree was constructed based on the chloroplast genomes of 19 published Campanulaceae species, utilizing two Asteraceae species as outgroups. The results indicated the following: (1) The chloroplast genome of *Pseudocodon convolvulaceus* is 183,616 bp in length, featuring a typical tetrad structure with a GC content of 38.7%. A total of 134 genes were annotated, comprising 89 protein-coding genes, eight rRNA genes, and 37 tRNA genes, with 12 genes containing one intron and three genes containing two introns. (2) The chloroplast genome includes 67 SSR loci, predominantly single nucleotide repeats, which account for 40% of the total. (3) The genome comprises 64 synonymous codons, including 30 high-frequency codons (RSCU > 1), with 29 of these high-frequency codons ending in A/T, representing 96.7%. This suggests a tendency for high-frequency codons in the chloroplast genome of *Pseudocodon convolvulaceus* to terminate with A/T. (4) Phylogenetic analysis revealed that *Codonopsis minima*, *Codonopsis lanceolata*, *Codonopsis pilosula*, and *Codonopsis tsinlingensis* are closely related to *Pseudocodon convolvulaceus*. The findings of this study enhance our understanding of the genetic basis of this species and its potential applications in drug research, thereby facilitating the use of this genomic resource for conservation strategies and phylogenetic analysis.

## 1. Introduction

Chloroplasts are the primary sites of photosynthesis in green plants and are classified as semi-autonomous organelles. They are widely distributed and primarily found in green plants and algae [1]. This organelle contains thousands of proteins that play crucial roles not only in photosynthesis but also in the synthesis of various biological molecules, including fatty acids, amino acids, hormones, vitamins, nucleotides, and secondary metabolites [2]. Chloroplasts possess a distinct set of genetic mechanisms, primarily represented by the chloroplast genome. In most plants, the chloroplast genome exhibits a characteristic tetrad structure, comprising two inverted repeat regions (IRs) with identical sequences but opposite orientations, flanked by a large single copy region (LSC) and a small single copy region (SSC) [3]. Due to its high abundance, highly conserved structure, single gene copy, minimal recombination, and relatively short full-length sequence, the chloroplast genome has emerged as a vital research tool in diverse fields, including phylogenetic analysis, genetic diversity assessment, phylogeography, germplasm resource identification, and molecular breeding [4,5]. With advancements in sequencing technology and a reduction in costs, the chloroplast genome has been extensively studied across various plant species. For instance, it has been utilized in phylogenetic analyses of *Arisaema takesimense* [6], *Erythrina variegata* L.[7], *Polygonatum zanlanscianense* [8], and other plants to elucidate their evolutionary dynamics, phylogenetic relationships, and potential applications in medicinal research. This study significantly enriches the available genomic resources and facilitates solving problems related to phylogenetic relationships and species identification.

*Pseudocodon* Wall. is a genus of the Campanulaceae family, all species of which are perennial herbs. There are approximately 70 species of *Pseudocodon* globally, with the majority found in Eastern and Central Asia. In China, approximately 39 species of *Pseudocodon* are distributed across the southwestern provinces and regions [9]. *Pseudocodon convolvulaceus* is a perennial twining herb primarily found in grasslands and pine forest understories at altitudes ranging from 2,000–3,000 meters in Southwest China. *Pseudocodon convolvulaceus* is a widely recognized Tibetan medicinal plant. Its tuberous roots and flowers are commonly used in traditional medicine to treat conditions such as the common cold, gastric ulcers, gastritis, anemia, spontaneous sweating, tracheitis, hernias, insufficient lactation, and neurasthenia [10]. Similar to other *Pseudocodon* species, the primary chemical constituents of *Pseudocodon convolvulaceus* are triterpenoids and sterols [11]. Due to its significant medicinal value, *Pseudocodon convolvulaceus* is extensively harvested by local people, resulting in a growing scarcity of wild resources. Furthermore, the distribution range of *Pseudocodon convolvulaceus* is limited, with a notably small population and poor resource regeneration capacity, leading to a shortage in market supply [12]. Therefore, there is an urgent need to investigate the genetic resources of this plant.

In recent years, the studies of the chloroplast genomes have been extensively applied to various species within the Campanulaceae family, including *Adenophora divaricata*, *Adenophora remotiflora*, *Codonopsis lanceolata*, *Codonopsis minima*, and *Campanula takesimana*. These studies provide valuable insights into the genetic structure of Campanulaceae plants. However, the limited research on the chloroplast genome of *Pseudocodon convolvulaceus* has impeded a comprehensive exploration and development of its potential applications. Consequently, this study aims to fulfill the need for chloroplast genome sequencing of *Pseudocodon convolvulaceus* to elucidate its gene composition, evolutionary significance, and medicinal potential, thereby enhancing the genomic resources available for the genus *Pseudocodon* and its phylogenetic relatives. By comparing its chloroplast genome with those of other closely related Campanulaceae species, this research seeks to highlight its unique genetic characteristics and provide a reference for future studies on plant phylogeny and conservation.

## 2. Materials and methods

### 2.1. Sample collection

**Fig 1:**
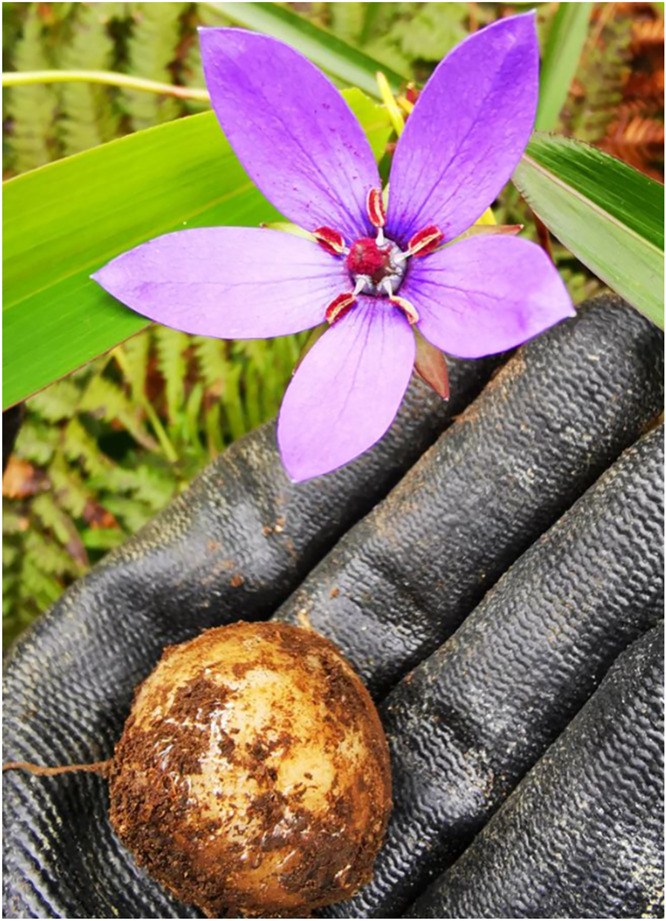
Photo of *Pseudocodon convolvulaceus*, taken at Nyingchi City, Tibet Autonomous Region. The roots are tuberous, nearly ovoid, 2.5-5 cm long, grayish yellow. Corolla spokeshaped and nearly 5-lobed, lobes elliptic, 1-2.5 cm long, pale blue or bluish-purple. Filament bases are broad and densely villous.

To investigate the systematic position and genetic background of *Pseudocodon convolvulaceus*, we sequenced the DNA of *Pseudocodon convolvulaceus* and obtained its complete chloroplast genome. The voucher specimen was collected from the grass slopes in Azga Village, Gongbujiangda County, Nyingchi City, Tibet Autonomous Region, China, on August 15, 2020 (3081.27 m, E93°31’3“, N29°53’52”) (Fig 1). Our samples have been confirmed by our agency (School of Life Sciences, Qinghai Normal University) and no additional licenses are required. The specimen has been deposited at the Herbarium of the School of Life Sciences, Zhengzhou University, with the voucher number ZZU2020–7686 (Mr. Zuo, 740886849@qq.com).

### 2.2. DNA extraction and sequencing

Total genomic DNA was extracted using a plant genomic DNA extraction kit (Solarbio LIFE SCIENCES, China, catalog number: [D1500]). DNA library preparation was performed using the NovaSeq 6000 S4 v1.5 kit (catalog number: [20028312]). A 150 bp paired-end library was prepared for sequencing.

### 2.3. Genome assembly and annotations

Using NOVOPlasty v4.2 (*https://github.com/ndierckx/NOVOPlasty*) [13], we assembled the chloroplast genome, with the comprehensive results serving as the final genome for screening. In addition to manual inspection, the newly assembled chloroplast genomes were annotated using PGA (Plastid Genome Annotator) [14], with the chloroplast genome of *Codonopsis tsinlingensis* (Genbank: NC_056284.1) serving as a reference. The tRNA genes were verified using ARAGORN v1.2.38 (*https://anaconda.org/bioconda/aragorn*) [15] and tRNAscan-SE v2.0.7 (*https://github.com/UCSC-LoweLab/tRNAscan-SE*) [16]. A completely annotated circular plastid map was created using OGDRAW (*https://chlorobox.mpimp-golm.mpg.de/OGDraw.html*) [17].

### 2.4. Comparative analysis of chloroplast genomes

Based on the chloroplast genome sequence and its annotation, we analyzed the fundamental characteristics of the chloroplast genome of *Pseudocodon convolvulaceus*, including genome size, gene content, and the number of various genes. We downloaded the chloroplast genome sequences of 19 species from the Campanulaceae family and two species from the Asteraceae family from the NCBI database (*National Center for Biotechnology Information*). The chloroplast genomes of 20 species from the Campanulaceae family and two species from the Asteraceae family, including *Pseudocodon convolvulaceus*, were compared using MAFFT v7.0 software (*MAFFT* - a multiple sequence alignment program) [18]. The mVISTA online tool (*http://genome.lbl.gov/vista/mvista/submit.shtml*) [19] was employed to visualize the overall similarity among the chloroplast genomes of these 20 Campanulaceae and two Asteraceae species, with annotations conducted in ShuffleLAGAN mode. Additionally, the IRScope online platform (*https://irscope.shinyapps.io/Chloroplot/*) [20] was utilized to analyze the genes at the boundary junctions of the four regions of the chloroplast genomes, assessing whether there were contractions or expansions in the IR region.

### 2.5. Identification of SSRs and repeat sequences

The MISA [21] *(https://webblast.ipk-gatersleben.de/misa/index.php)* online tool was employed to identify simple sequence repeats (SSRs) with a minimum sequence length of 10 bp. The threshold for the minimum number of repeats was set at 10 for single nucleotides, five for dinucleotides, and four for trinucleotides. For tetranucleotides, pentanucleotides, and hexanucleotides, the minimum repeat count was established at three. Furthermore, the distance between any two SSRs was required to be at least 100 bp. Finally, redundant repetitive sequences were removed, and the prediction results were manually refined.

The online software REPuter(*https://bibiserv.cebitec.uni-bielefeld.de/reputer*) [22] was used to identify four types of potential repetitive sequences within the chloroplast genome: forward repeats, reverse repeats, palindromic repeats, and complementary repeats. The minimum length was established at 30 bp, the Hamming distance was set to three, and the similarity of the repeat sequences was required to exceed 80%.

### 2.6. Analysis of codon usage

The protein-coding genes of the *Pseudocodon convolvulaceus* chloroplast genome were extracted using PhyloSuite software [23]. To prevent bias in codon usage calculation, a full-length examination was conducted for each protein-coding gene to exclude genes shorter than 300 bp or those with non-ATG start codons [24]. CodonW v1.4.4 software was used to calculate the number of codons and the relative synonymous codon usage (RSCU) for all protein-coding genes. An RSCU value greater than one indicates that the codon is a high-frequency codon and is frequently used, whereas an RSCU value less than one signifies the contrary.

### 2.7. Phylogenetic inference

We selected the complete chloroplast genomes of 21 species closely related to *Pseudocodon convolvulaceus* for phylogenetic analysis, some of which have been previously studied [25–27]. It has been reported that numerous rearrangements have occurred in the chloroplast genomes of the Campanulaceae family [28]. Therefore, we utilized Geneious Prime 2022.2.2 to extract only the protein-coding genes (PCGs) from the chloroplasts for our phylogenetic analysis. The chloroplast genome of *Pseudocodon convolvulaceus* and 21 chloroplast genome sequences, downloaded from GenBank, were aligned using MAFFT [29]. Subsequently, phylogenetic trees were constructed using the maximum likelihood (ML) method in MEGA X, with 1,000 bootstrap replicates [30]. *Centaurea maculosa* (Asteraceae) and *Centaurea diffusa* (Asteraceae) were selected as outgroups.

## 3. Results

### 3.1. Basic characteristics of the chloroplast genome of *Pseudocodon convolvulaceus*

The complete chloroplast genome of *Pseudocodon convolvulaceus* is 183,616 bp in length and has been released to the NCBI database (GenBank accession no. NC_060685.1). This genome comprises two inverted repeats (IRs) of 38,454 bp, which are separated by a large single copy (LSC) region of 96,234 bp and a small single copy (SSC) region of 10,474 bp. The overall GC content of the chloroplast genome is 38.7%, with the corresponding values for the LSC, SSC, and IR regions being 37.6%, 34.3%, and 40.7%, respectively. Notably, the complete chloroplast genome of *Pseudocodon convolvulaceus* is significantly longer than those of other *Pseudocodon* species. For instance, the complete chloroplast genomes of *Codonopsis tsinlingensis*, *Codonopsis pilosula*, and *Codonopsis minima* are 170,253 bp, 170,672 bp, and 169,321 bp in length, respectively.

**Fig 2:**
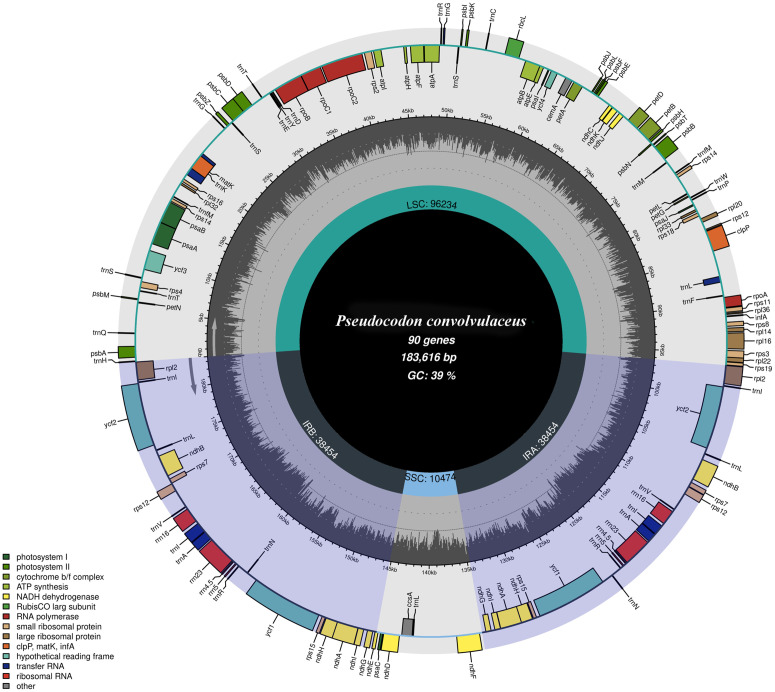
The complete chloroplast genome map of *Pseudocodon convolvulaceus.* Arrangement of 136 genes represented in the map, including 91 protein-coding genes, 37 tRNA genes, and eight rRNA genes. The GC% along the chloroplast is represented by the inner circle.

The chloroplast genome of *Pseudocodon convolvulaceus* consists of 134 genes, which include 89 protein-coding genes, eight rRNA genes, and 37 tRNA genes (Fig 2). Among these, 24 genes are duplicated (*ndhA, ndhB, ndhG, ndhH, ndhI, rpl2, rps12, rps14, rps15, rps7, rrn16, rrn23, rrn4.5, rrn5, trnA-UGC, trnI-CAU, trnI-GAU, trnL-CAA, trnN-GUU, trnR-ACG, trnV-GAC, trnfM-CAU, ycf1, ycf2*). Twelve genes possess a single intron (*ndhA, ndhB, petB, petD, atpF, rpl16, rpl2, trnA-UGC, trnG-UCC, trnI-GAU, trnK-UUU, trnL-UAA*). Additionally, three genes (*rps12, clpP, ycf3*) contain two introns (Table 1).

**Table 1:** Gene composition of the chloroplast genome of *Pseudocodon convolvulaceus.*

Category for gene	Gene group	Gene name
Photosynthesis	photosystem I	*psaA,psaB,psaC,psaI,psaJ*
	photosystem II	*psbA,psbB,psbC,psbD,psbE,psbF,psbH,psbI,psbJ,psbK,psbL,psbM,psbN,psbT,psbZ*
	Subunits of NADH dehydrogenase	ndhA*(2),ndhB*(2),*ndhC,ndhD,ndhE,ndhF,ndhG*(2),*ndhH*(2),*ndhI*(2),*ndhJ,ndhK*
	Subunits of the cytochrome b/f complex	petA,petB*,petD*,*petG,petL,petN*
	Subunits of ATP synthase	atpA,atpB,atpE,atpF*,*atpH,atpI*
	Large subunit of Rubisco	*rbcL*
	Subunits of photochlorophyllide reductase	–
Self-replication	Proteins of the large ribosomal subunit	rpl14,rpl16*,rpl2*(2),*rpl20,rpl22,rpl32,rpl33,rpl36*
	Proteins of the small ribosomal subunit	rps11,rps12**(2),*rps14*(2),*rps15*(2),*rps16,rps18,rps19,rps2,rps3,rps4,rps7*(2),*rps8*
	Subunits of RNA polymerase	*rpoA,rpoB,rpoC1,rpoC2*
	Ribosomal RNAs	*rrn16*(2),*rrn23*(2),*rrn4.5*(2),*rrn5*(2)
	Transfer RNAs	trnA-UGC*(2),trnC-GCA,trnD-GUC,trnE-UUC,trnF-GAA,trnG-GCC,trnG-UCC*,*trnH-GUG,trnI-CAU*(2),trnI-GAU*(2),trnK-UUU*,*trnL-CAA*(2),trnL-UAA*,*trnL-UAG,trnM-CAU,trnN-GUU*(2),*trnP-UGG,trnQ-UUG,trnR-ACG*(2),*trnR-UCU,trnS-GCU,trnS-GGA,trnS-UGA,trnT-GGU,trnT-UGU,trnV-GAC*(2),*trnW-CCA,trnY-GUA,trnfM-CAU*(2)
Other genes	Maturase	*matK*
	Protease	clpP**
	Envelope membrane protein	*cemA*
	Acetyl-CoA carboxylase	–
	C-type cytochrome synthesis gene	*ccsA*
	Translation initiation factor	*infA*
	other	–
Genes of unknown function	Conserved hypothetical chloroplast ORF	*ycf1*(2),*ycf2*(2),ycf3**,*ycf4*

Notes: Gene*: Gene with one introns; Gene**:Gene with two introns; #Gene: Pseudo gene; Gene(2):Number of copies of multi-copy genes.

### 3.2. Chloroplast genome function and classification

Based on gene function, the 134 genes annotated in the chloroplast genome of *Pseudocodon convolvulaceus* can be categorized into four groups (Table 1): 49 genes associated with photosynthesis, 74 genes involved in self-replication, five genes with other functions, and six genes of unknown function.

### 3.3. Contraction and expansion of inverted repeats

**Fig 3:**
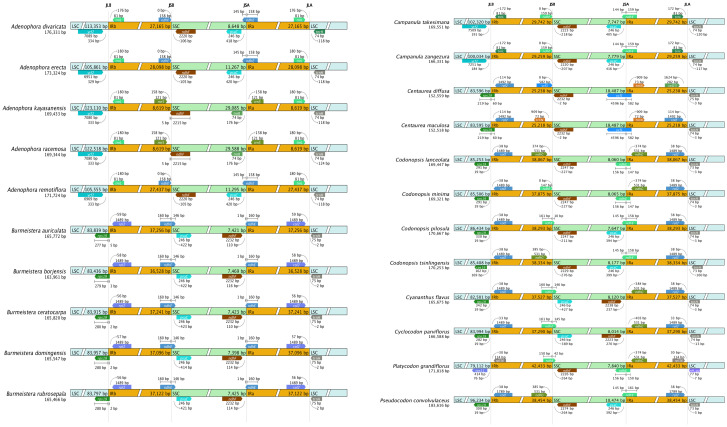
Comparison of the regional boundaries of the chloroplast genomes of 20 species of Campanulaceae and two species of Asteraceae.

Due to the contraction and expansion of the IR region, the size of the chloroplast genome may vary, which in turn affects the evolutionary rate of chloroplast genes. A comparison of the chloroplast genomes of 20 species within the Campanulaceae family and two species from the Asteraceae family revealed that the most pronounced IR boundary positions were located at IRb/SSC and SSC/IRa (Fig 3). The *ndhE* gene, located at the boundary between the JSB and JSA regions, crosses from the IRb and IRa regions into the SSC region, except for two Asteraceae species and two Campanulaceae species (*Adenophora racemosa* and *Adenophora kayasanensis*). The LSC/IRa boundary is relatively conserved, with the *rpl2* gene situated in the IRa at the junction of the LSC and IRa, except for one species from the Asteraceae family (*Centaurea diffusa*), three species from the Campanulaceae family (*Platycodon grandiflorus*, *Campanula takesimana*, and *Campanula zangezura*), as well as *Adenophora*. The *trnL* gene in *Adenophora* is located at the junction of LSC and IRa in IRa. The *trnH* gene is located in LSC at the junction of LSC and IRa, except in *Platycodon grandiflorus* and *Adenophora divaricata*. The LSC/IRb boundary is relatively conserved. In certain species, the *rps19* gene is situated within the LSC region at the junction of the LSC and IRb, effectively crossing the LSC/IRb boundary and entering the LSC region from the IRb region. In most species, the junction of the LSC and IRb for the *rpl2* gene is located within the IRb region. Additionally, the *ndhE* gene, situated at the JSA boundary of *Pseudocodon convolvulaceus*, crosses from the IRa region into the SSC region.

### 3.4. Codon usage bias analysis

**Fig 4:**
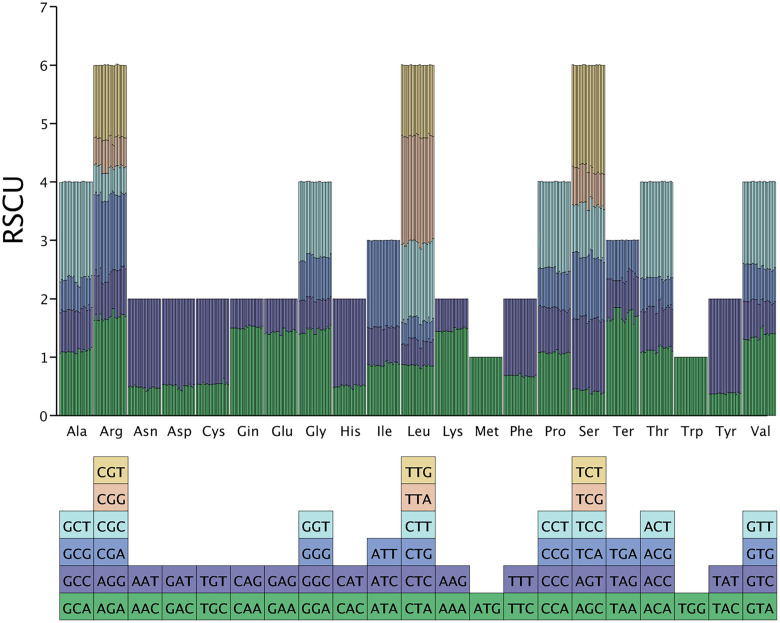
RSCU codon bias.

**Fig 5:**
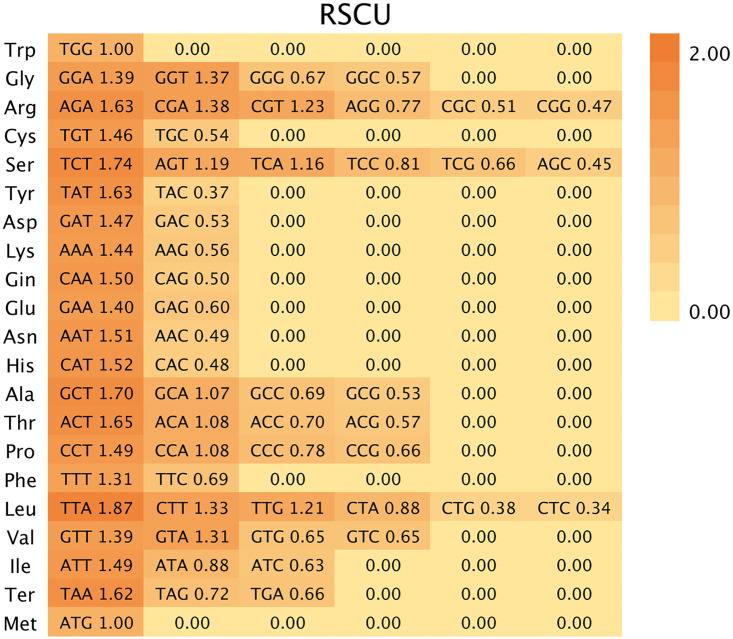
Codon RSCU value.

The chloroplast genome of *Pseudocodon convolvulaceus* contains 21 amino acids and 64 codons. Except for Met and Trp, which utilize only one codon each (ATG and TGG, respectively), the remaining amino acids exhibit between two to six synonymous codons (Fig 4). The number of high-frequency codons, defined as those with a relative synonymous codon usage (RSCU) greater than one, totals 30, accounting for 46.88% of the overall codons. Notably, 29 of these codons end with A/T bases, suggesting a preference for A/T terminations in the chloroplast genome of *Pseudocodon convolvulaceus*. 32 low-frequency codons showed an RSCU value of less than one, which accounted for 50% of the total codons. Among these, 29 codons ended with G/C bases, indicating a tendency for low-frequency codons in the chloroplast genome of *Pseudocodon convolvulaceus* to conclude with G/C. Additionally, there were nine codons with an RSCU greater than 1.5. Notably, Leu showed a preference for the TTA codon, which had the highest RSCU value of 1.87. Ser followed closely, with an RSCU value of 1.74, demonstrating a bias toward the use of TCT codons (Fig 5).

### 3.5. Comparative analysis of chloroplast genomes

The mVISTA method was employed to further compare the chloroplast genomes of *Pseudocodon convolvulaceus*, revealing a similar sequence pattern across the entire chloroplast genome. This analysis included 20 species from the Campanulaceae family and two outgroup species: *Centaurea diffusa* and *Centaurea maculosa*.

**Fig 6:**
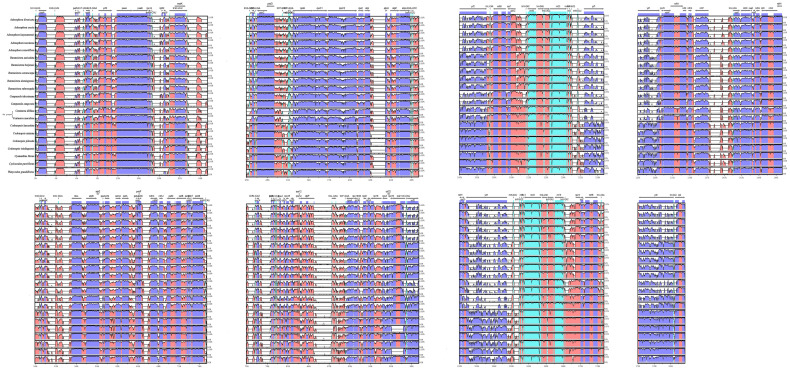
Comparison of the chloroplast genome structure between 20 species of Campanulaceae and two species of Asteraceae.

The results indicated that, in comparison to the two outgroups, the overall similarity of the chloroplast gene sequences among Campanulaceae species was greater (Fig 6). Furthermore, variations in the non-coding regions were observed, with the LSC and SSC regions exhibiting a higher degree of variation than the IR region. There were significant differences in the coding regions of *ycf1*, *ycf2*, *ycf3*, *rpoC2*, *rpoC1*, *rpoB*, *rpoA*, *rps2*, *rps3*, and *ndhF* in the chloroplast genome. Additionally, there were significant differences in *rps4*-*ycf3*, *clpP*-*rps14*, *atpH*-*atpF*, *trnfM-CAU*-*trnK-UUU*, *trns-GCU*-*trnC-GCA*, *trnL-UAA*-*trnF-GAA*, *rps12*-*trnV-GAC*.

### 3.6. SSR and repetitive sequence analysis of chloroplast genomes

**Fig 7:**
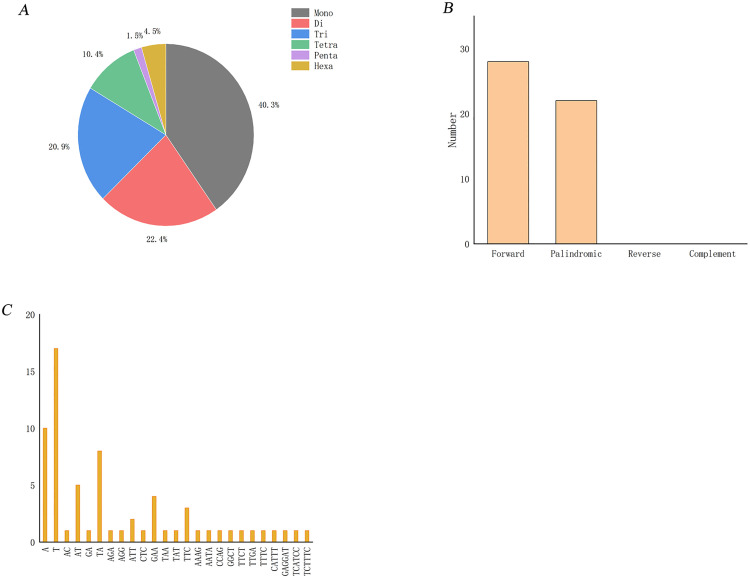
SSRs and repeats of chloroplast genome of  *Pseudocodon convolvulaceus. * A. The number of different SSRs; B: The number of four replicates; C: Type and number of repeat bases of SSR‌.

A total of 67 SSRs were detected in the chloroplast genome of *Pseudocodon convolvulaceus*, of which single nucleotide repeats were the most, accounting for 40% of the total; Dinucleotide, trinucleotide, tetranucleotide, pentanucleotide and hexanucleotide repeats accounted for 22%, 21%, 10%, 2% and 5%, respectively (Fig 7A). From a base composition perspective, SSR loci predominantly consist of A, T, or AT complex repeats, suggesting a strong preference for A and T in the chloroplast genome of *Pseudocodon convolvulaceus* (Fig 7C). The analysis of repeat sequences revealed that the number of forward repeats is the highest in the chloroplast genome of *Pseudocodon convolvulaceus*, followed by palindromic repeats, while no reverse or complementary repeats were detected (Fig 7B).

### 3.7. Analysis system development

**Fig 8:**
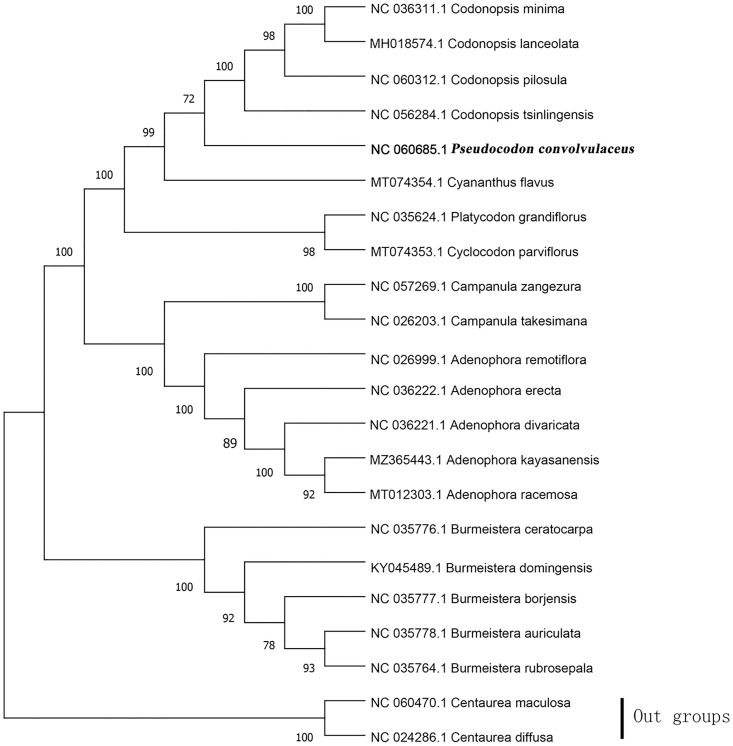
Phylogenetic tree of 22 species based on only protein-coding genes of the chloroplast genome using the ML (with 1000 replicates) method. The numbers below the branches indicate the corresponding bootstrap support values from the ML tree.

The results of the phylogenetic analysis showed that Campanulaceae and Asteraceae each formed a monophyletic clade. Five *Burmeistera* species, five *Adenophora* species, and two *Campanula* species within the Campanulaceae each formed a monophyletic clade with 100% support. *Platycodon* and *Cyclocodon* form a monophyletic clade with 98% support. *Pseudocodon convolvulaceus* clustered in the same clade with *Codonopsis minima*, *Codonopsis lanceolata*, *Codonopsis pilosula*, *Codonopsis tsinlingensis*, and *Cyananthus flavus*, indicating that they are closely related. Compared to *Burmeistera*, *Adenophora* formed a monophyletic clade with *Campanula*, indicating a closer phylogenetic relationship; *Platycodon*, *Cyclocodon*, together with *Pseudocodon convolvulaceus*, *Codonopsis minima*, *Codonopsis lanceolata*, *Codonopsis pilosula*, *Codonopsis tsinlingensis*, and *Cyananthus flavus*, collectively formed a monophyletic clade. Furthermore, within the Campanulaceae, the seven genera *Pseudocodon*, *Codonopsis*, *Cyananthus*, *Platycodon*, *Cyclocodon*, *Campanula*, and *Adenophora* together formed a monophyletic clade, which was completely distinct from *Burmeistera* and showed a closer phylogenetic relationship among themselves (Fig 8).

## 4. Discussions

In this study, the chloroplast genome sequence characteristics of *Pseudocodon convolvulaceus* were analyzed from multiple perspectives. The results indicated that the chloroplast genome structure of *Pseudocodon convolvulaceus* is similar to that of other species within the same genus, as well as to those from different genera in the Campanulaceae family, all of which exhibit a typical circular tetrad structure. In this study, we identified a total of 134 genes encoded by *Pseudocodon convolvulaceus*, which includes 89 protein-coding genes, eight rRNA genes, and 37 tRNA genes. The complete chloroplast genome measures 183,616 bp and exhibits a GC content of 38.7%. Notably, the chloroplast genome of *Pseudocodon convolvulaceus* is longer than that of most other known species within the Campanulaceae family. For instance, the total genome length of *Campanula takesimana*, a closely studied plant, is 169,551 bp [31]. Additionally, the total genome lengths of the three closely related species *Codonopsis minima* [25], *Codonopsis tsinlingensis* [32], and *Codonopsis lanceolata* [33] are 169,321 bp and 170,253 bp, and 169,447 bp, respectively. Chloroplast microsatellite sequences have emerged as crucial genetic markers for studying adaptive evolution and genetic diversity within species, owing to their uniparental inheritance characteristics and high levels of polymorphism. These sequences are frequently utilized as essential molecular markers in genetic diversity analyses and the identification of closely related species [34,35]. A total of 50 repetitive sequences and 67 SSR loci were identified in the chloroplast genome of *Pseudocodon convolvulaceus*, with single nucleotide repeats of A/T being the predominant type. This finding further corroborates that SSRs in the chloroplast genome are primarily composed of polyadenine (poly A) and polythymine (poly T) [36]. These results are consistent with those observed in the chloroplast genomes of *Platycodon grandiflorus* and *Adenophora remotiflora* [37,38]. The detected repeat sequences provide a valuable reference for further studies on SSR markers, molecular genetics, and crop breeding of *Pseudocodon convolvulaceus*.

The IR region within the chloroplast genome of plants is recognized as the most conserved region. Variations in the contraction and expansion of its boundary region sequences can result in alterations in the copy number of associated genes or the formation of pseudogenes within this boundary region [39]. Notably, the types of flanking genes and the degree of expansion of the tetrad boundary in *Pseudocodon convolvulaceus* exhibit significant differences compared to those found in other species of the Campanulaceae and Asteraceae families. The *ndhE* gene of *Pseudocodon convolvulaceus*, located on the JSA boundary, crosses the boundary from the IRa region into the SSC region. These differences result in the generation of pseudogenes, gene duplication and gene deletion. For instance, Kyeong-Sik Cheon et al. have demonstrated that the IRb/SSC boundary extends into *ndhE* to create a *ndhE* pseudogene in the chloroplast genomes of *Campanula takesimana*, *Adenophora remotiflora*, *Adenophora erecta* and *Adenophora divaricata* [27]. The chloroplast genome sequences of plants are frequently utilized as molecular markers for DNA barcoding in species identification. In this study, the genes *ycf1*, *ycf2*, *rpoC2*, and *ndhF* exhibit significant variability and may serve as potential indicators for species identification and research on genetic diversity. Current studies indicate that *ycf1* is an effective marker for assessing the phylogenetic relationships among *Adenophora* species [27].

Codon usage bias refers to the tendency of organisms to preferentially select specific codons throughout long-term evolution [40]. The results of this study indicate that the chloroplast genome of *Pseudocodon convolvulaceus* exhibits a preference for ending with A/T, which aligns with the characteristics of the chloroplast genomes observed in certain terrestrial plants [41]. According to the results of the phylogenetic tree construction, among the 20 selected species of Campanulaceae, *Codonopsis tsinlingensis*, *Codonopsis minima*, *Codonopsis lanceolata*, and *Codonopsis pilosula* were found to be the closest relatives to *Pseudocodon convolvulaceus*. Notably, *Codonopsis minima* constituted the most basal lineage within the Campanulaceae branch. The support rate for this finding was 100%, aligning with the research conclusions of Kyeong-Sik Cheon and Huijuan Zhou et al.[25,32]. *Adenophora racemosa* is most closely related to *Adenophora kayasanensis*, which is consistent with the findings of Kyung-Ah Kim et al.[42]. *Burmeistera* formed a monophyletic clade, while the two Asteraceae species are grouped into a separate clade, aligning with current taxonomic classifications.

## 5. Conclusions

In summary, this study completed the sequencing and assembly annotation of the chloroplast genome sequence of *Pseudocodon convolvulaceus*. It revealed the fundamental characteristics of the *Pseudocodon convolvulaceus* chloroplast genome and the codon usage bias at the gene level. This work lays an important theoretical foundation for subsequent studies on the genetic structure and genetic diversity of Campanulaceae plants. In addition, based on the complete chloroplast genome coding sequences of 22 species, this study further analyzes the phylogenetic relationships among Campanulaceae and Asteraceae, as well as between *Pseudocodon* and *Codonopsis*, *Cyananthus*, *Platycodon*, *Cyclocodon*, *Campanula*, *Adenophora*, and *Burmeistera*. This analysis provides a scientific basis for the systematic classification of Campanulaceae *Pseudocodon*. Therefore, the results of this study provide fundamental data for future research, including the development of molecular markers for Campanulaceae plants, as well as studies on the phylogeny, genetic diversity, germplasm screening and utilization of *Pseudocodon convolvulaceus*.
